# Embryonic and extraembryonic tissues during mammalian development: shifting boundaries in time and space

**DOI:** 10.1098/rstb.2021.0255

**Published:** 2022-12-05

**Authors:** Shifaan Thowfeequ, Shankar Srinivas

**Affiliations:** Department of Physiology, Anatomy and Genetics, University of Oxford, South Parks Road, Oxford OX1 3QX, UK

**Keywords:** extraembryonic tissues, history of embryology, fate specification, fate commitment, mammalian development, embryonic-extraembryonic boundaries

## Abstract

The first few days of embryonic development in eutherian mammals are dedicated to the specification and elaboration of the extraembryonic tissues. However, where the fetus ends and its adnexa begins is not always as self-evident during the early stages of development, when the definitive body axes are still being laid down, the germ layers being specified and a discrete form or bodyplan is yet to emerge. Function, anatomy, histomorphology and molecular identities have been used through the history of embryology, to make this distinction. In this review, we explore them individually by using specific examples from the early embryo. While highlighting the challenges of drawing discrete boundaries between embryonic and extraembryonic tissues and the limitations of a binary categorization, we discuss how basing such identity on fate is the most universal and conceptually consistent.

This article is part of the theme issue ‘Extraembryonic tissues: exploring concepts, definitions and functions across the animal kingdom’.

## 1. Introduction

The *afterbirth* whose name reflects the time of its emergence is also, all too often, an afterthought, except perhaps to embryologists. It exists because humans and other eutherian mammals are matrotrophic viviparous amniotes [1]. As a result, they have evolved specialized transient structures to support the nutritional, respiratory and excretory needs of the fetus. Such structures also provide mechanical and immunological protection during fetal development within the uterus of the mother [2,3]. These so-called ‘extraembryonic’ tissues are the first to emerge and differentiate well before the development of any fetal precursors is initiated. As development progresses, the extraembryonic tissues and those of the fetus gradually become more anatomically distinct, but early during development until their individual fates are determined, these boundaries are not as concrete.

### (a) Why we need to define embryonic-extraembryonic boundaries

Agreed terminology and their unambiguous definition are critical in ensuring clarity when discussing concepts. For developmental biologists, this is all the more important, to avoid confusion and misunderstanding, especially in an age when artificial ‘embryos’ and embryonic components can be generated *ex vivo* [4–10]. An appreciation of how specific terminology came into being is also important for interpreting the wealth of information in historic texts that form the foundation for modern developmental biology. Further, it can be relevant to philosophical discussions on the individuality of the embryo and helps to anchor what might otherwise be metaphysical definitions of our individuality in empirical facts of early embryonic development [11,12]. Here, we will outline how the way in which we define the boundaries (both categorical and anatomical) between the *embryonic* and *extraembryonic* has shifted throughout history, both within developmental space and through developmental time. In the light of current knowledge, we will try to reach a logical agreement on what these terms should encompass, while calling attention to inevitable exceptions and attempting to address how they can be best accommodated.

### (b) What constitutes an extraembryonic tissue?

**Figure 1.**
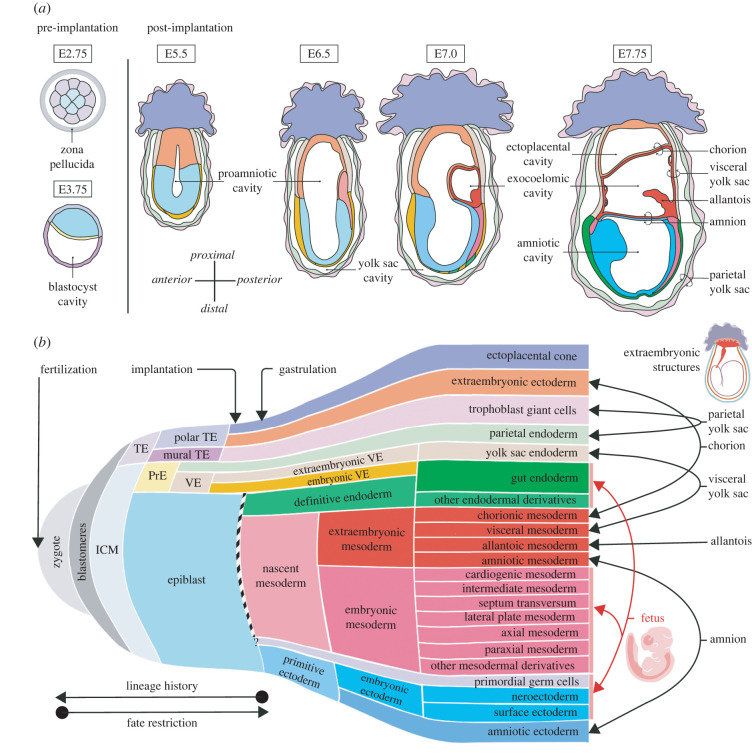
(*a*) The changing morphology and tissue composition of the mouse conceptus. After implantation, the conceptus is surrounded by maternal tissues (not shown). The intraembryonic cavities and the extraembryonic structures are labelled. Use (*b*) as a key for tissue identities. (*b*) The sequential emergence of embryonic and extraembryonic tissues of the conceptus starting from the fertilized egg. Cell fate commitment of TE-derivatives is seen first leading up to and in preparation for implantation, while those of epiblast-derivatives arise following gastrulation. The black-and-white vertical line demarcate the primitive streak and the subsequent fate of cells that ingress through it. The extraembryonic membranes are primarily bilayers while the fetus itself is made up of three germ layers (ectoderm, mesoderm and endoderm) and germ cells. The embryonic or extraembryonic status of any tissue can be assessed by tracing its fate forwards through developmental time (to the right) and seeing if it contributes to embryonic component or only extraembryonic structures. (TE, trophectoderm; ICM, inner cell mass; PrE, primitive endoderm; VE, visceral endoderm.)

The term ‘embryo’ generally refers to all tissues arising from the fertilized egg, up until an anatomically distinct fetus containing all the organ primordia of the future individual is identifiable, at around mid-gestation. This however generates the inconsistency of having to describe extraembryonic tissues as being part of the early embryo which then leads to awkward usages such as ‘embryo proper’ when referring to that subset of tissues that gives rise to the fetus. To avoid this, the term conceptus has been used in reference to all derivatives of the zygote in their entirety, both those that give rise to the fetus that is born, as well as those that contribute to extraembryonic tissues lost at birth (figure 1*a,b*).

**Figure 2.**
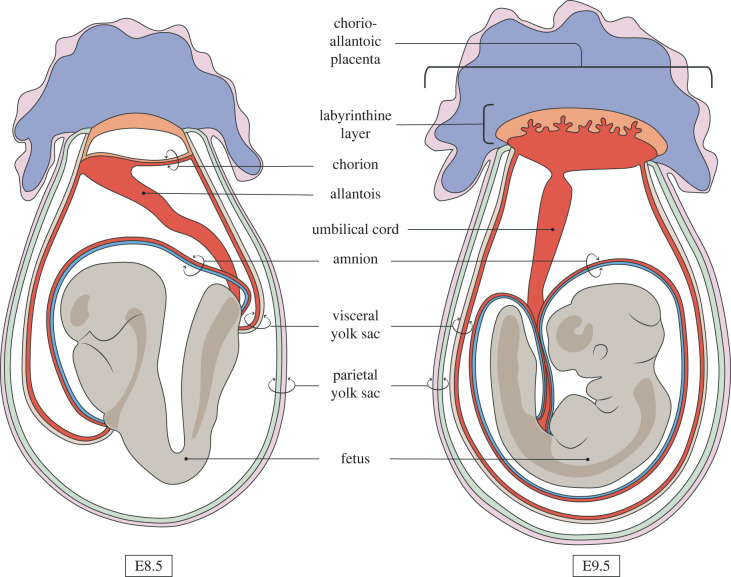
The position of extraembryonic structures relative to the mouse fetus, before (E8.5) and after (E9.5) embryo turning. While the amnion and visceral yolk sac surround the fetus, the allantois and chorion invade the ectoplacental cone, to give rise to the chorio-allantoic placenta. From then on, the amnion, visceral yolk sac, the placenta and umbilical cord persist until the end of gestation.

**Figure 3.**
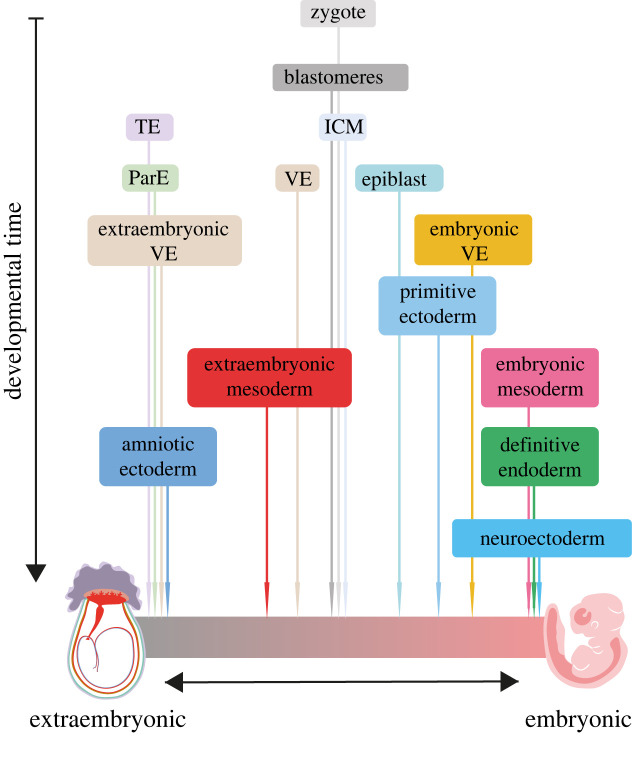
Illustration of the concept of an embryonic-extraembryonic continuum onto which all tissues of the developing embryo can be mapped. Only selected tissues are shown as examples. Tissues are initially composed of cells with both embryonic and extraembryonic potential, and they gradually move towards either extreme, depending on the proportion of their constituent cells with particular fates. Examples of exclusively embryonic or extraembryonic tissues are not as common as previously thought. (TE, trophectoderm; ICM, inner cell mass; ParE, parietal endoderm; VE, visceral endoderm.)

The categorization of tissues as extraembryonic has been made based on anatomical location, histology, functional differences or on the basis of molecular markers. Given the defining feature of extraembryonic tissues is that they are zygotically derived but do not contribute to the fetus, we suggest that ultimately, it is the fate of cells within a tissue that should be paramount in designating it as extraembryonic or not. In this review, giving fate precedence, we will explore concepts of extraembryonic and embryonic tissue identity, using the terms according to the following definitions, while highlighting any exceptions to the rule:

– an **embryonic** tissue is one whose fate is to contribute to *any* structure that is retained in the fetus, and by extension, becomes part of the born ‘individual’,
– an **extraembryonic** tissue is one whose fate is to primarily give rise to those structures that support the embryo during its development (e.g. the placenta, extraembryonic membranes, umbilical cord; figures 2 and 3) and not retained as part of the individual after birth.

With several examples covering stages of development from conception to beyond gastrulation, we will put this binary definition to the test. We will inquire whether such distinct tissues fated to exclusively contribute to either the fetus or its adjoining extraembryonic structures, even exist within the early embryo. As cell commitment, and then determination, is a gradual process, cells progressively restrict their range of fates towards becoming embryonic and/or extraembryonic tissues (figure 1*b*) [13]. As a result, whether a tissue or its descendants is categorized as embryonic or extraembryonic can continually shift across developmental time—cells destined to extraembryonic fates are derived from progenitors or precursors that could be categorized as embryonic. This makes it important therefore to always keep in mind the developmental stage under discussion when considering whether a tissue is ‘embryonic’ or ‘extraembryonic’. Technological advances involving lineage tracing techniques in the mouse (e.g. [14]) and discerning the epigenetic characteristics of cells that presage fate restriction (eg: [15]) currently allow us to more confidently base our definition in fate than ever before, irrespective of developmental stage.

## 2. Changing notions of the extraembryonic through history of embryology

Through history, the designation of extraembryonic structures shifted from being based on their speculated function, anatomical location and then morphological characteristics, before their origins and fates could be well characterized. These changes in the way we think about what constitutes the extraembryonic part of the conceptus closely mirrors the history of scientific rationale, following the advances in methodology and technological tools available to study embryonic development in increasingly finer detail.

### (a) From a mystery to anatomical structures with functions

Through antiquity and across cultures, the afterbirth was entrenched in mysticism, folklore and superstition. The first theory of the placenta as being an organ of fetal nutrition is attributed to Diogenes of Appollonia (*ca* 480 BC), while Hippocrates (*ca* 460–370 BC) subscribed to the standard view at the time that the fetus nourished itself *per os* within the uterus by suckling on placental cotyledons [16–18]. Aristotle (384–322 BC) was the first to study anatomy by systematic observation, identifying that the fetus was enclosed within membranes and nourished directly from maternal blood via the umbilical cord ([19], Trans.). He also identified the chorion and allantois (see figures 1 and 2) but incorrectly postulated their origins to lie in coagulated male and female semen (*flora alba*), respectively, an idea propagated by the other notable anatomist of antiquity, Claudius Galenus (Galen of Pergamum; *ca* AD 130–201; [16]). In Western intellectual thought, the Aristotelian and Galenic views were held as doctrine for more than a millennium. Owing to the wealth of literature available and its accessibility, the history of embryology might seem a purely European pursuit up until modern times. However, it is worth noting that the flux of ideas between Europe and Asia through history influenced embryological thought on both sides and curiosity about the origin of the embryo existed independently across a multitude of cultures [18,20–22].

Much of the study of embryology during the Renaissance and the Age of Enlightenment was dominated by the debate on the extent to which maternal and fetal bloods were interconnected. It was finally resolved by William Harvey (1651; [23] in [24]) establishing that fetal and maternal circulation were separate, an idea elaborated by Marcello Malpighi (1661; [25]) and later validated by Camillis Falconet (1752; [26]). Harvey also recognized blood islands at the periphery of the early embryo as antecedents to the heart (1653; [27]). By then, it had been known that the placenta consisted of both maternal and fetal tissue [28], and that the chorion was essential for implantation and placentation (Hunter, 1774, 1794; [29,30]).

### (b) Microscopy and the age of descriptive embryology

Even at the dawn of the age of microscopy, many authors shared contemporary beliefs in preformation, which had authoritarian enforcement at the time. This idea held that all organisms come into being as preformed miniatures of themselves, leaving little room to debate the origin of extraembryonic structures [31]. However, unrelenting advances in our understanding such as the discovery of the mammalian egg (von Baer, 1827; [32]) and resolving the mystery of fertilization (Newport & Forbes, 1854; [33]) argued in favour of epigenesist thought (note that the use of the term ‘epigenesis’ in neoclassical embryology is distinct from the use of ‘epigenetics’ in developmental biology today; see [34]) that development progresses through the elaboration of form in an unstructured zygote (Remak, 1860; [35]), with every structure having an origin (lineage) and a destiny (fate).

By the nineteenth century, it became commonplace to study the nature of tissues based on their morphological characteristics through careful microscopic observations and not just their anatomical location [31]. For example, the early suggestions that the chorion, owing to its superficial location, arose from the corona radiata of the ovum (von Baer) were replaced by recognition that it was a double continuous layer and was of fetal origin (Langhans, 1870; [36]). To meet the pedagogic demands of the time, a dizzying array of new terminology entered the discipline, often with a multiplicity of terms for the same tissue depending on the species it was described in [37]. As advances in compound microscopy facilitated the possibility of charting the course of these newly described tissues during development, a gradual shift in thinking about the origin and fate of tissues also became apparent.

### (c) Mapping the origin and fate of tissues

Hubrecht (1889; [38]) identified for the first time a portion of the blastocyst that did not contribute to the formation of the fetus and named it the trophoblast [39]. The term trophectoderm (TE; see figure 1*a*) was only later adopted (borrowing from its use in the marsupial unilaminar blastocyst, [40]) to distinguish between these superficial cells before and after implantation. The peri-implantation embryo was also studied in great detail leading to distinguishing between tissues with a common origin [41], such as the parietal and visceral entoderm (later endoderm; see figure 1*a* and following section for details) initially based on marked histological distinctions [42,43].

Questions about how the fate of cells in mammalian embryos was determined remained unanswered well into the twentieth century. Microenvironmental differences in position were linked to fate determination of blastomeres, with the formulation of the ‘inside-outside’ hypothesis, where cells occupying an outside position within the morula were fated to become extraembryonic [44,45]. During the latter half of the twentieth century, through the generation of chimeras, and, disaggregation and reaggregation approaches, the lineal origins of many post-implantation tissues were connected to their pre-implantation precursors [46,47]. Further refinement of these methods allowed fate to be determined, by transferring cells heterotopically (between locations) or heterochronically (between stages) or by labelling cells and following their progeny [48–52]. One such seminal study using DiI-labelling of cells within the pre-gastrulation embryo led to the discovery of a subset of cell necessary for its anterior–posterior axial patterning (see following section for details), for the first time assigning a true ‘instructive’ function to a tissue thought of as being ‘only’ extraembryonic [53]. Advances in genetic manipulation of the mouse, robust transgenic reporters to trace specific lineages, and high-resolution time-lapse imaging approaches to visualize genetically-labelled cell populations in real time, together revolutionized research into the origin and fate of extraembryonic tissues over the next decades [54–56]. Today, extraembryonic tissues are not merely structures confined to a specific anatomical location, possessing a distinct histological character or expressing a specific molecular marker, but they can be identified based on the changing potential of cells, as their fates are gradually restricted and ultimately determined.

## 3. The sequential emergence of extraembryonic tissues during development

In the following sections, we elaborate on the conceptual framework of a definition based on fate, using specific examples from the early conceptus, where commitment to extraembryonic fates is made progressively by cells with embryonic potential.

### (a) Pre-implantation derivation of extraembryonic tissues

Blastomeres of the mouse morula are morphologically indistinguishable from each other and totipotent, and even at the 16- and 32-cell stage, individual cells have the capacity to give rise to both embryonic and extraembryonic tissues [57]. The emergence of the first exclusively extraembryonic tissue can be seen with the formation of the blastocyst, and the differentiation of the TE, an extraembryonic tissue that encloses the embryonic inner cell mass (ICM) in eutherian mammals (see [58], to compare with the first lineage allocations in non-eutherian mammals). In the late blastocyst of the mouse, the TE maintains its extraembryonic fate as it differentiates into the polar and mural TE, gearing up for implantation (figure 1) [59,60]. During this time, the ICM differentiates into two distinct tissues—the primitive endoderm and the epiblast [61]. One of the derivatives of the primitive endoderm, the parietal endoderm is the next tissue to acquire an exclusively extraembryonic fate, as its cells migrate to line the mural TE to form the parietal (or primary) yolk sac.

### (b) Diversification and convergence of endodermal lineages

Following implantation, the other derivative of the primitive endoderm, the visceral endoderm (VE; analogous to the hypoblast in other mammals), remains in close association with the pluripotent embryonic epiblast. Great morphogenetic diversity is seen between different mammalian taxa during the process of epiblast specification [62]. Until gastrulation, the parietal endoderm and VE facilitate nutrient and waste exchange between the implanted conceptus and the maternal tissues. In murid rodents, where the peri-implantation conceptus goes on to acquire an atypical cup shape, the VE differentiates over the proximal and distal regions, with distinct morphological and molecular characteristics [63]. The proximal region, which lines the polar TE-derived extraembryonic ectoderm, is a cuboidal epithelium that is continuous with the parietal endoderm, and likewise contributes to the yolk sac (figure 1*a*). By contrast, the distal region surrounding the epiblast is largely squamous. Within this latter region, a subpopulation of cuboidal cells emerges and migrates to the prospective anterior to form the anterior VE (AVE; [64]). AVE migration sets the polarity of the anterior–posterior axis of the conceptus and restricts the site of gastrulation to the opposite side of the egg cylinder [65]. Morphologically and transcriptionally analogous cells to the AVE have been described within the hypoblast of monkey embryos [66,67] and cultured human embryos [68], as well as those that are functionally equivalent to the AVE, in rabbit embryos [69].

During gastrulation, epiblast cells exit pluripotency to commit to one of three principle germ layers of the fetus—ectoderm, endoderm or mesoderm. The default fate of epiblast cells is to contribute to ectodermal derivatives [15]. Epiblast cells at the posterior, disengage from the adjacent embryonic VE, to form a transient space, the primitive streak. The modified basement membrane lining this region allows epiblast cells to delaminate and ingress through the primitive streak following an epithelial-to-mesenchymal transition [70–72]. Towards the anterior/distal region of the primitive streak, *FoxA2*-expressing cells re-epithelialize giving rise to the definitive endoderm (DE) which then intercalates with the underlying embryonic VE to collectively form the precursor of the gut tube (figure 1*b*) [54,73]. Therefore, in addition to the morphological and molecular differences between the distal and proximal regions of the VE mentioned above, they also seem to have distinct embryonic and extraembryonic fates, respectively.

### (c) Mesodermal contribution to extraembryonic structures

In the mouse, the mesoderm is generated during gastrulation as a population of *Brachyury (T)*-expressing cells that ingress through the primitive streak [74]. Some of these cells migrate anteriorly along the mesodermal wings to form the embryonic mesoderm, which diversifies and contributes to various mesodermal structures of the fetus (figure 1*b*). Another subpopulation of these nascent mesodermal cells migrates proximally to accumulate adjacent to the extraembryonic ectoderm and becomes the extraembryonic mesoderm, fated to contribute to various extraembryonic structures [75,76]. Here, a new cavity, the exocoelom, forms in between these cells (figure 1*a*). As this cavity expands, at its distal extreme, extraembryonic mesoderm along with the adjacent ectoderm of the epiblast undergo morphogenetic remodelling to converge at the anterior, bisecting the proamniotic cavity into the amniotic and ectoplacental cavities, with the newly formed amnion as the partition separating them [77,78]. Extraembryonic mesoderm cells also line the extraembryonic ectoderm and extraembryonic VE surrounding the exocoelemic cavity, forming the chorion and visceral (also secondary or definitive) yolk sac, respectively [79]. The allantois buds off from the junction of the visceral yolk sac and the amnion, and grows diagonally across the exocoelemic cavity towards the chorion with which it fuses [80–82]. The final arrangement of the amniotic membrane and yolk sac surrounding the fetus results from cephalocaudal and lateral folding, and turning, reversing the topology of the embryo (figure 2). Parts of the allantois, chorion and visceral yolk sac are then incorporated with derivatives of the ectoplacental cone and with each other to form the chorio-allantoic placenta and the umbilical cord (figure 2) [79,83,84]. During subsequent developmental stages, the placenta in eutherian mammals combines into one discrete organ many of the physiological functions that are postnatally divided among the body's various organ systems.

Similar to the primitive ectoderm, the nascent mesoderm of the mouse arises as an embryonic tissue, from which distinct extraembryonic components are subsequently derived following gastrulation. By contrast, there is evidence that the extraembryonic mesoderm of primates, including humans, originates soon after implantation as an extraembryonic tissue that contributes to primary yolk sac formation before gastrulation [85,86]. Its developmental origin is the subject of extensive debate, with some sources rooting it in the hypoblast while others had previously suggested the trophoblast [87]. Nevertheless, unlike in the mouse, in primates, the initial extraembryonic mesoderm arises from *GATA4/6-*positive*, T-*negative cells [88], although it is probably later supplemented by mesoderm from an embryonic epiblast origin generated at gastrulation [89,90]. Another difference between primates and the mouse is that the amniotic cavity in primates is formed by the cavitation of the epiblast, leading to the formation of the amniotic ectoderm prior to gastrulation. Taken together, it is evident that the sequence of events leading to the formation of extraembryonic tissues from their embryonic precursors varies between species and is primarily dependent upon where and when their fates are determined.

## 4. Comparison of fate with other commonly used characters for defining embryonic-extraembryonic identity

Here, we compare some of the other criteria on the basis of which cells and tissues have been categorized as extraembryonic and identify problems and inconsistencies associated with them.

### (a) Anatomy

As we have seen, the embryonic or extraembryonic status of tissues was historically designated based on speculated supportive functions assigned to them depending on their anatomical location relative to the fetus. These notions were long held, as embryonic-extraembryonic fate distinction among groups of cells usually follow anatomical boundaries, with the more exterior/superficial cells (TE, parietal endoderm, VE) of the conceptus adopting an extraembryonic fate, while those encased within (ICM, epiblast) go on to contribute to the fetus. This is even more pronounced after gastrulation when the fetus is recognizable as an anatomically distinct entity (around nine days in mice and eight weeks in humans) with rudimentary precursors to all the body structures, surrounded by extraembryonic membranes and connected to the placenta and maternal tissues by the umbilical cord (figure 2).

However, there are several exceptions to this rule. The embryonic-extraembryonic boundary is ever-changing, as cells that go on to occupy the fetal portion of the conceptus can have extraembryonic origins. In both rodents and primates, blood islands within the mesodermal walls of the yolk sac function as the first observable sites of erythropoietic activity [91]. The yolk sac-derived primitive blood enters into embryonic circulation and resides within the anatomical confines of the fetus during mid-gestation development [92]. Succeeding waves of definitive erythropoiesis is taken on by embryonic organs (aorta-gonad-mesonephros, fetal liver and bone marrow), and these cells go onto contribute to definitive anucleate erythrocytes that perdure postnatally (see, [93]). Therefore, early embryonic blood is extraembryonic, both in origin and fate, despite its transient anatomical location within the fetus.

Another example is the fate of the embryonic VE in the mouse. It was long thought that the epiblast-derived DE inserts itself into the VE as a continuous sheet, displacing the VE so that it could only contribute to extraembryonic structures. However, genetic labelling and careful time-lapse tracking of cell movements revealed that DE cells get interspersed among the superficially located embryonic VE cells, together with which they contribute to the inner lining of the gut tube [54]. Later, single-cell RNA sequencing analyses showed that the transcriptome of VE-derived cells of the gut endoderm converge with that of epiblast-derived DE descendants while maintaining transcriptional signatures of their origin [73,94]. Whether this transcriptional heterogeneity based on lineal origin results in biases in fate within organ primordia of the fetus or stem cell niches in the adult remains to be established.

### (b) Molecular identities

Even before transcriptomic approaches, single or multiple marker gene expression was widely used as a criterion to distinguish between embryonic and extraembryonic tissues. Such gene expression patterns can be used to demarcate the boundaries between tissue types at a given time, but do not necessarily remain unchanged as development progresses. For example, the extraembryonic TE can be distinguished from the embryonic ICM of the early blastocyst by the expression of *Cdx2* in the former and *Oct4* in the latter [95,96]. However, during development, the same genes are often reused in different tissue types, in close succession to each other. *Cdx2* is in fact regulated by two distinct *cis*-enhancers in the TE and its derivative, the extraembryonic ectoderm, with *in vitro* TE-derived trophoblast stem cells being more similar to extraembryonic ectoderm in this respect [97]. Therefore, the degree to which blastocyst-derived stem cells (embryonic stem (ES), trophoblast stem and extraembyonic stem cells) can recapitulate their respective precursors can vary and *in vitro* differentiation trajectories do not always mimic those *in vivo*. Similarly, the homeobox gene *Hex* which is a marker of the AVE is also expressed in the anterior DE and later on in development in endothelial precursors, the liver and thyroid primordia, with early and late expression being driven by distinct enhancers [98]. The T-box transcription factor Eomesodermin, which is expressed in the extraembryonic ectoderm prior to gastrulation is later recruited transiently by extraembryonic mesoderm of the yolk sac as a regulator of hematopoietic development, as well as various embryonic mesodermal cell types [99,100]. These examples reiterate the importance of exercising caution when assigning tissue identity based on the expression of single or even several marker genes, especially in the context of organoids or ES and induced pluripotent stem cell-derived cells *in vitro*. With no precise reference frame in developmental time, it is not always straightforward to determine which *in vivo* state a cell generated *in vitro* best represents. Differentially expressed genes no doubt could play roles in demarcating and maintaining embryonic and extraembryonic boundaries, as shown by the differential expression of Ephrin/Eph family members in tissues of the mouse peri-implantation conceptus [63]. Nonetheless, single markers, while indicating the ‘state’ of a cell, themselves are not adequate for determining the fate of a cell.

In the past decade, single-cell transcriptomic approaches have aimed to overcome this obstacle by first assigning identities to transcriptomic clusters using marker gene expression, but then extracting a transcriptomic signature for the cells, capturing within it the expression states and levels of many genes [94,101]. This can be a vital tool in comparing *in vitro*-derived cells to their *in vivo* counterparts [89,102]. Such approaches have revealed how classic marker genes often span transcriptomic cluster boundaries and greatly vary in level of expression within clusters [94,103]. Advances in spatial-transcriptomics will enable us to better determine the extent to which transcriptomic boundaries correspond with anatomical boundaries [104], which is especially useful in characterizing cells in transitional regions where anatomical boundaries meet.

Single-cell transcriptomic approaches provide a snapshot view of the transcriptomes of cells in the midst of constant flux, undergoing the gradual process of committing to more definitive fates. Such snapshots, from multiple cells collected at different stages of development, can be used to infer the *in vivo* ontogeny of tissues [105]. In the mouse for example, as the AVE migrates, it becomes transcriptionally more distant from the embryonic VE from which it originates, than the embryonic VE is from the extraembryonic VE [63]. It would therefore be interesting to see how the fate of AVE cells differs from that of other VE cells contributing to the gut endoderm as a consequence of this inferred transient transcriptomic divergence. Such computational inferences can provide important insights, but need to be verified experimentally.

Multiomic approaches such as scNMT-Seq (a method for the parallel profiling of chromatin accessibility, DNA methylation and the transcriptome from single cells) have also proved to be powerful in determining what might be considered the ‘default’ state of cells and their immediate fate, based on their epigenetic potential [15]. However, with datasets covering limited windows of developmental time, it can be challenging to determine the embryonic or extraembryonic status of cells, as it has to take into consideration all subsequent fate choices made by the cell up until birth. Designating fate using classic approaches such as generating chimeras still remains the most robust approach, especially when testing the potential of *in vitro* generated tissues to contribute to embryonic or extraembryonic lineages [106]. Where such approaches or genetic intervention is not possible, as with human embryos, lineage histories can be drawn by mapping somatic mutations or tracking mitochondrial heteroplasmy among cells, which when interpreted in reverse (forward in developmental time) can be indicative of fate specification [107,108].

### (c) Cellular morphology and behaviour

Another means by which embryonic and extraembryonic distinctions have been made in the past is by comparing the morphology of tissues and the constituent cells. Similar to how fate choices often follow anatomical boundaries, the same can be true for histomorphological boundaries. For example, in the mouse, the primitive endoderm-derived VE, though forming a continuous epithelium, segregates into columnar extraembryonic and squamous embryonic regions (figure 1*a*). Abrupt morphological boundaries are also seen in the transition between the extraembryonic amniotic ectoderm and embryonic surface ectoderm and neuroectoderm later in mouse development (fig. 2*a* from [109]) or between the amniotic and embryonic ectoderm in pre-gastrulation human embryos (fig. 23 on plate 3 from [110]). However, such clear-cut distinctions are not universal. For example, the intercalating DE cells are morphologically indistinguishable from the existing VE cells, which is one of the reasons why an appreciation VE contribution to the gut endoderm evaded us for so long. Similarly, in pre-gastrulation primate embryos, the extraembryonic mesoderm is continuous with, and aside from a few ultrastructural features, morphologically indistinguishable from, the primary yolk sac endoderm, making their origin harder to determine.

The structural properties of a cell and their surrounding extracellular matrix, could also modulate their ability to communicate with adjacent and underlying cells, which in turn could affect their behaviour and fate. The AVE, derived from the embryonic VE, acquires a columnar morphology more similar to their extraembryonic counterparts, yet they are behaviourally very distinct in their ability to actively migrate. Taking just histomorphology into consideration could lead to misleading conclusions on the embryonic or extraembryonic status of these cells. Differences in cellular behaviour can also be seen in mesodermal cells upon leaving the primitive streak. While the trajectory of embryonic mesodermal cells is more direct, that of the extraembryonic mesoderm tends to be more convoluted [111]. Molecular heterogeneities very likely underpin some of behavioural differences between these cells, but the extent to which this is a consequence of intrinsic epigenetic determinants or extrinsic factors arising from the surrounding microenvironment is unknown.

### (d) Comparison between species

Much of our understanding of the development of extraembryonic tissues in primates is based on experiments done in the mouse. Although embryogenesis in primates is largely similar to that in rodents, differences in topology of the conceptus, initial origin of the extraembryonic mesoderm, and the formation of the embryonic cavities remind us of the difficulty in assigning embryonic or extraembryonic identity to tissues based on commonalities in anatomical location or histomorphology between taxa. Evolutionarily conserved organization of extraembryonic tissues is seen among various amniotes during early development, but their topographical organization can diverge depending on the availability of nutritional supplies or how it is accessed [112]. For example, the very early specification of a presumptive extraembryonic tissue, the TE, is a uniquely eutherian characteristic closely related to its mode of development, wherein robust extraembryonic structures need to be established for invasive implantation and proper chorio-allantoic placentation. In their sister group, the marsupials, the pluriblast, which occupies a similar peripheral location within the blastocyst to that of the TE, remains a mixed population of cells with both embryonic and extraembryonic potential until much later in development [113]. Taking this into consideration, when considering diverse mammalian taxa with different embryonic organizations and reproductive strategies, there is more reason to base embryonic and extraembryonic status of tissues on species-specific fate and not homology—a point which was emphasized more than three decades ago [114].

It would be interesting to see in the future how the differences in the order of emergence of extraembryonic tissues between species correlates with types of placentation, lengths of gestation and the nutritional demands of the fetus—all features highly reliant on the extraembryonic tissues themselves.

## 5. Important considerations when using a definition grounded in fate

In this section, we consider some of the challenges of using an operational definition based on fate. We will highlight some examples that challenge a simple binary categorization of tissues as extraembryonic or embryonic, and propose how these can be reconciled, based on the fate of the constituent cells of those tissues.

### (a) When is fate determined?

The main challenge in assigning tissue identity based on fate is ascertaining when fate is determined, since fate can be more restricted than potential. Fate is what a particular cell gives rise to within a specific time frame of reference, while potential is all the cell types it is capable of giving rise to. Uncommitted cells show gene expression changes in response to specific intrinsic and extrinsic cues that allow them to start to differentiate towards a specific fate. In such a state of restricted potential, the fate of the cells can be said to be *specified*. These changes are however labile and can be reversed. Further gene expression changes accentuate these early differences and irreversibly (during normal development and under non-pathological conditions) seal the fate of cells. The fate of the cells is then said to be *determined*.

Fate, by definition, cannot be ascertained from a snapshot of developmental time. A cell might express a repertoire of genes indicative of being capable of differentiating down a specific trajectory, but if it is exposed to new signals (as consequence, of changing position within the embryo for instance), its prospective fate could be altered.

For example, biases might exist as early as the 2- or 4-cell stage in the ability of each blastomere to contribute to different proportions of embryonic (ICM) and extraembryonic (TE) lineages [115,116]. However, these are not necessarily inherent determinants of fate and the biases can be reversed if the blastomere positions were to change. More recent system-based approaches have shown indications of how heterogeneity between blastomeres of the 8-cell conceptus could prime them for early fate specification but this is unlikely to represent determination [117]. As such, the equivalence in potential of blastomeres is best demonstrated by cases of monozygotic polyembryony among mammals, where blastomeres could split prior to blastocyst formation, giving rise to separate conceptuses with their own embryonic and extraembryonic derivatives. Similarly, the recent use of somatic mutations to infer clonal relationship between cells has shown that there can be a great deal of variability between individuals in the extent to which the early 2-celll blastomeres contribute to the adult [118,119]. Even after compaction, aggregates made entirely of inside or outside cells can form normal blastocysts and within the ICM, individual cells are initially indistinguishable from each other at a transcriptomic level [120]. The stochastic transcriptomic heterogeneities between blastomeres or the cells of the ICM are gradually amplified and reinforced by epigenetic changes leading to fate determination [117,120]. Identifying the modifications to the epigenome that are responsible for restricting the potential of different extraembryonic tissues at each branch point (figure 1*b*) may help us pinpoint when the fate choices are made.

Distinguishing between specified and determined fate is even more challenging in later stages of development. Lineage tracing studies with inducible reporters can help map the fate of cells labelled at a specific time. Despite the technical challenges of such experiments in mammals, heterotypic transplantation of cells between different regions and stages of mouse egg cylinders has also been performed to show that the allocation of cells to the various mesodermal lineages is dependent on the timing of their ingression through the primitive streak, with their potency becoming restricted as gastrulation progresses [121]. Although the first cells to traverse the primitive streak contribute to extraembryonic lineages, cells from the pre-streak epiblast transplanted among the mid-streak epiblast do not do so [121]. This suggests that the fate of cells in the epiblast is not irreversibly determined prior to gastrulation but rather, that they respond to cues from the surrounding cells in committing to the options available at a given time. It is unclear however if epiblast cells are in some sense already committed prior to traversing the streak, or whether the timing of their ingression through the streak positions them in different regions of the embryo that provide different inductive cues that determine their fate. Studying whether these progenitors already bear epigenetic marks prior to gastrulation, indicative of their fate, could help resolve this question. However, it will be important to also establish whether such epigenetic marks are irreversible.

Given that some mammalian groups such as primates have evolved mechanisms to generate extraembryonic mesoderm independent of gastrulation [87], such approaches might help explain the mechanisms whereby the pre- and post-gastrulation extraembryonic mesoderm cells in these species transcriptionally and presumably functionally converge to contribute to the same extraembryonic organs. This may occur in an analogous way to how the DE and VE, despite their distinct developmental origins, converge to form the foregut endoderm of the fetus [73,94].

### (b) Cells crossing embryonic-extraembryonic boundaries

Determining the fate of cells at the boundary between tissue types and transitional zones is often complicated, largely because it is often unclear where these boundaries precisely lie, and whether they are strict or somewhat blurry. Such boundaries include, for example, the embryonic-extraembryonic VE boundary, the anterior endoderm furrow after the amniotic ectoderm makes its connection, the connecting stalk of the allantois, and the Juxta Cardiac Field (JCF) at the confluence of splanchnic, somatic and extraembryonic mesoderms [103]. Cells in these zones might be more capable of switching fates depending on their exact positions. Detailed imaging studies [111] and potential future spatial epigenomics technologies [122] to visualize epigenetic marks indicative of fate commitment in an anatomical context might help us to more clearly define the nature and degree of porosity of such boundaries.

The finer the detail in which we study embryogenesis, the more we see situations where tissues we speak of as extraembryonic, actually harbouring cells that also contribute to the fetus. For example, although yolk sac-derived primitive blood is transient, as discussed earlier, the yolk sac-derived hematopoietic cells could go on to colonize the hepatic primordia and contribute to the very early stages of fetal liver haematopoiesis [123]. Similarly, myeloid progenitors also emerge from the yolk sac mesoderm and give rise to macrophages that go on to populate the developing brain and become microglia. As the blood–brain barrier forms, these cells expand within the confines of the central nervous system and are maintained into adulthood [124,125]. Cells of the allantois can also differentiate into definitive erythroid and myeloid lineages *in vitro*, and this might be indicative of such differentiation occurring *in vivo* [126,127]*.* The proximal portion of the allantois is incorporated into the fetus during caudal folding and the formation of the primitive urogenital sinus, that later gives rise to the urinary bladder. This connection of the forming bladder with the allantois narrows until it ultimately degenerates to form a structure called the urachus [128]. After birth, it is retained as a dense fibrous structure, the median umbilical ligament [129]. A final example is that of the previously described JCF—an embryonic tissue contributing progenitor cells to the developing heart [103]. Based on its location at an embryonic-extraembryonic junction with boundary-spanning gene expression profiles, careful study is warranted to rule out any extraembryonic contribution to this cell population.

These examples challenge a binary categorization of tissues, as cells within many of these tissues possess the potential to contribute to different fates and are highly migratory. If considering tissue at the level of the individual component cells, there are perhaps few tissues that can be recognized as exclusively embryonic or extraembryonic until much later in development. Therefore, an additional perspective we could introduce is to consider embryonic or extraembryonic identity as constituting opposite extremes of a continuum. The different tissues of the developing conceptus can then be mapped along this continuous measure depending on the proportion of their constituent cells restricted to one fate or the other (figure 3). Such a framework ultimately would also allow us to accommodate the spatially blurry and temporally changing nature of embryonic-extraembryonic boundaries. In this framework, the fate of a tissue as a whole would remain undefined until the fates of *all* its constituent cells are known. As far as we know, it is tissues such as the TE or the parietal endoderm that do not contribute any cells to the fetus, that can be categorized as extraembryonic much earlier compared to tissues such as the extraembryonic mesoderm or the VE.

## 6. Concluding remarks

For effective scientific discourse, it is of utmost importance that there is clarity in definitions and the terminology used. We suggest that despite its technical challenges, basing embryonic and extraembryonic categorization of tissues on the fate of their constituent cells is the most conceptually consistent approach. The history of scientific ideas closely mirrors the history of scientific methods. At a time when new technologies allow us to study embryonic development with ever finer spatial, temporal and molecular resolution in a steadily increasing range of models, we recall that ‘our real teacher has been and still is the embryo—who is incidentally the only teacher who is always right’ (Viktor Hamburger, see [130, p. xi]). We will have to be prepared to respond to new findings by regularly re-evaluating the categories we impose on tissues in the developing conceptus without being hindered by preconception.

## Data Availability

This article has no additional data.
